# Caries of Vertebræ

**Published:** 1872-06-15

**Authors:** Daniel Lichty

**Affiliations:** Rochelle, Ill.


### CARIES OF VERTEBRÆ.

BY DANIEL LICHTY, M.D., OF ROCHELLE, ILL.

This case is presented because of its rarity as regards age and its peculiar and varied symptoms.

Mr. Pott, in his diseases of the spine, says he never saw caries occur at an age beyond forty, and Mr. Baynton never met with more than three instances which approached that period of life, while Mr. Brodie, in his Diseases of Joints, describes one case in which the patient was forty-five years old; and Sir Astley Cooper *thinks* he had a patient near that age.

Mr. O. F. aged 40, average size and erect figure, marked bilious temperament, without any malignant hereditary predisposition, though all his family bear this strongly marked bilious type; occupation, farmer, applied at my office Feb. 16, 1872, for advice, when he presented the following history, symptoms and signs: Has for two years been troubled with slight indigestion; during two years past had what farmers style a "crick in the back," and thirteen months before consulting, was severely hurt in right side by the horn of a cow, but no physician being consulted, the extent and character of the injury was never known, yet he said he never fully recovered from it; work in the stooping posture, violent exercise, riding in a farm wagon, each had to be occasionally abandoned from the pain which they created, while an interval of rest gave him relief; still he continued managing and working his farm until February last. He presented some slight nervous trepidation, rose from his chair cautiously; could not bear any weight on his hand when extended at right angles to his body; rheumatic pains in loins, constant but not excited on pressure; the perfect free mobility of back somewhat impaired; pressure in right hypochondrium referred to right scapula; a sense of fullness in epigastrium with anorexia, while percussion and palpation showed positive enlargement of the liver; this organ protruding from beneath the ribs to the extent of nearly an inch, while the middle lobe could be traced to the umbilicus and to the left of the median line; skin and sclerotica both jaundiced. Diagnosed, chronic hepatitis with mal-assimilation of nerve substance and consequent nervous irritation. Alteratives—hydrg. chlor. mit. sanguinaria and pot. nit. were first employed; being unusually susceptible, slight plyalism occurred. Iodide and bromide of potassium followed this with external irritants and absorbents, which reduced the size of the liver and appeared to establish its functions; the pain in epigastrium disappeared; skin and sclerotica became clear and of a natural appearance, and appetite returned. Tonic medicines—phosphate of iron, strychnia, etc., with nutritious food I hoped would now restore him. Valuable counsel had been called to my aid, and the foregoing diagnosis and treatment confirmed.

But here my anticipations began to falter; instead of recovering he kept on gradually growing more excitable and weaker, though contrary to what subsequent developments proved, he could until a late period of his illness walk up and down stairs; but the slightest misplacement of body would bring on a cramp or spasm which he described as feeling as though his back was bent double; by bending the third and second phalanges of his finger upon the first. Here he became unable to walk, and was confined to his bed, with occasional rest in the sitting posture with the body thrown well forward and supported over the back of a chair. Atrophy of the spinal cord, caries of the spine and kindred maladies were suspected but none could be detected; not the slightest unnatural curvature or prominence was observable; tapping or pressure would not elicit pain, and hot cloths applied gave him more relief than anything employed locally; the electro-magnetic current passed through every part of his body without any further result than to produce a moderate degree of excitement, never any wincing at any part of the spine; sensation in lower extremities not so acute as in trunk and arms; never any spasms referred to the thighs while co-ordination of motion remained perfect in legs; venery, though without intercourse, was natural; urination under control, while the tendency to constipation was relieved by two pills improv. cath. U. S. D. taken at 9 o'clock p.m.; decubitus was always on either side, never on the back; morbid and peculiar nervous sensations emanated from the sensorium and prevailed over the upper part of the body, marked by extreme hyperesthesia so that the hem of his shirt, or a wrinkle in the sheet could not be tolerated from the pressure they induced; the touch of a finger to his sides he could not allow; application in thought, or, to him, repulsive sights, would bring on spasms. At a period six weeks before his death the attendant could not move in his chair, scarcely sigh, unless patient was soundly asleep. In this interval of four weeks two able physicians saw him with me and he was unhesitatingly pronounced hypochondriacal by both.

Bromides controlled, in a measure, his excitability, while hydrate of chloral procured for him sleep at night. His appetite remained good, and intellection equal to any one of an equal length of sickness.

Twenty-four hours before his death symptoms of acute spinal meningitis attacked him from which he died.

The friends were persuaded from the obscurity of the case to allow a post mortem, which was held the day of his death. The lungs, liver, spleen and right kidney presented a healthy appearance, the left kidney showing signs of quite recent inflammation and engorgement; the mucous membrane of stomach inflamed at cardiac region; the white fibrous fascia binding the vertebrae was unimpaired, but pressure showed softness, and the scalpel penetrated the bodies of the vertebra and a mass of putrescence and bone spiculæ, the bodies of the vertebræ, from the sacro-lumbar articulation upward, all being carious and suppurated to the seventh cervical, some of them retaining their outline by the outer lamina only, many of them being eroded to the supervening cartilage. The form of disintegration indicated that it commenced anteriorly in the bodies, but at what point could not be ascertained; no point could be detected at which pus escaped nor were there at any time symptoms of pus escaping into the cavity of body or of abscesses. I think it one of those rare cases of which Sir Astley Cooper says of carious spine without curvature and without collections of matter forming and making their way outward, but "being detained within the body, destroys the patient, the REAL and IMMEDIATE *cause of whose death is seldom known, or even rightly guessed at, unless the dead body be examined*," and to that end submit it to the consideration of the profession.

